# Exercise therapy, education, and cognitive behavioral therapy alone, or in combination with total knee arthroplasty, in patients with knee osteoarthritis: a randomized feasibility study

**DOI:** 10.1186/s40814-024-01470-y

**Published:** 2024-02-28

**Authors:** Turid Rognsvåg, Ingvild Buset Bergvad, Ove Furnes, Kari Indrekvam, Anners Lerdal, Maren Falch Lindberg, Søren T Skou, Jan Stubberud, Mona Badawy

**Affiliations:** 1 Department of Orthopedic Surgery, Haukeland University Hospital, Coastal Hospital in Hagevik, Bergen, Norway; 2 Department of Clinical Medicine, University of Bergen, Bergen, Norway; 3 Department of Surgery, Lovisenberg Diaconal Hospital, Oslo, Norway; 4 Department of Interdisciplinary Health Sciences, Faculty of Medicine, Institute of Health and Society, University of Oslo, Oslo, Norway; 5 The Norwegian Arthroplasty Register, Department of Orthopedic Surgery, Haukeland University Hospital, Bergen, Norway; 6 Department of Research, Lovisenberg Diaconal Hospital, Oslo, Norway; 7 Department of Public Health Science, Faculty of Medicine, Institute of Health and Society, University of Oslo, Oslo, Norway; 8 Research Unit for Musculoskeletal Function and Physiotherapy, Department of Sports Science and Clinical Biomechanics, University of Southern Denmark, Odense, Denmark; 9 The Research Unit PROgrez, Department of Physiotherapy and Occupational Therapy, Næstved-Slagelse-Ringsted Hospitals, Slagelse, Denmark; 10 Department of Psychology, University of Oslo, Oslo, Norway

**Keywords:** Knee osteoarthritis, Physical exercises, Cognitive behavioral therapy, Feasibility trial

## Abstract

**Background:**

One in five patients experience chronic pain 1 year after total knee arthroplasty (TKA), highlighting the need for enhanced treatment strategies to improve outcomes. This feasibility trial aimed to optimize the content and delivery of a complex intervention tailored to osteoarthritis (OA) patients at risk of poor outcome after TKA and assess the feasibility of initiating a full-scale multicenter randomized controlled trial (RCT).

**Methods:**

Patients scheduled for TKA were included between August 2019 and June 2020 and block-randomized into one of three groups: (a) 12-week exercise therapy and education (ExE) and 10-module internet-delivered cognitive behavioral therapy (iCBT), (b) TKA followed by ExE and iCBT and (c) TKA and standard postoperative care. Outcomes were (i) recruitment and retention rate, (ii) compliance to the intervention and follow-up, (iii) crossover, and (iv) adverse events, reported by descriptive statistics.

**Results:**

Fifteen patients were included in the study. Only 1 out of 146 patients screened for eligibility was included during the first 4 months. During the next 3 months, 117 patients were not included since they lived too far from the hospital. To increase the recruitment rate, we made three amendments to the inclusion criteria; (1) at-risk screening of poor TKA outcome was removed as an eligibility criterion, (2) patients across the country could be included in the study and (3) physiotherapists without specific certification were included, receiving thorough information and support. No patients withdrew from the study or crossed over to surgery during the first year. Nine out of 10 patients completed the ExE program and six out of 10 completed the iCBT program. Fourteen out of 15 patients completed the 1-year follow-up. One minor adverse event was registered.

**Conclusions:**

Except for recruitment and compliance to iCBT, feasibility was demonstrated. The initial recruitment process was challenging, and necessary changes were made to increase the recruitment rate. The findings informed how a definitive RCT should be undertaken to test the effectiveness of the complex intervention.

**Trial registration:**

The MultiKnee RCT, including the feasibility study, is pre-registered at ClinicalTrials.gov: NCT03771430 11/12/2018.

## Key messages regarding feasibility

- 1) Uncertainties existed regarding the recruitment of patients and compliance with the intervention.
- 2) Challenges regarding recruitment were identified and improved during the feasibility study.
- 3) Revision of the iCBT program was needed to increase compliance with the intervention.

## Background

Hip and knee osteoarthritis (OA) is among the major causes of disability in the elderly population. The prevalence of OA is expected to increase due to increasing obesity and the aging population [1] indicating the importance of optimizing treatment options. The Osteoarthritis Research Society International (OARSI) guidelines state that the first-line treatment for knee OA includes education and structured exercise programs with or without dietary weight management. If non-surgical care is not sufficiently effective in terms of improving pain and function, it is recommended to refer patients to knee replacement surgery [2]. Total knee arthroplasty (TKA) surgery is a well-documented treatment for patients with moderate to severe knee OA. Most patients report very good clinical outcomes with improvement in pain, physical function, and quality of life [3, 4]. Projected estimates show an increasing demand for TKA for the treatment of OA with a steady increase in national registries [5, 6]. However, studies show that as many as 20% of patients undergoing TKA still have pain and poor function 1 year following surgery [7–9], leading to an increased number of revision procedures [10]. Psychological factors, such as catastrophic thinking [11], poor mental health [12], anxiety [13], and depression [14], have been associated with poor results after TKA. Skou et al evaluated the effectiveness of adding TKA to a combined non-surgical treatment program including education and exercise therapy. Even though the patients who received TKA experienced greater improvement than those without TKA, both groups experienced clinically relevant improvements in pain, function, and quality of life. Only 26% and 32% of patients who received education and exercise therapy alone had decided to undergo TKA at the 12- and 24-month follow-up, respectively [4, 15] suggesting that it is possible to reduce willingness to undergo surgery through engagement with guideline-recommended first-line care.

Exercise therapy and physical activity are also recommended in the rehabilitation after surgery [16]. There are uncertainties about to what degree individual patients adhere to these recommendations. Patients experiencing anxiety, depression, and catastrophic thinking regarding physical activity may have problems performing a prescribed exercise program [17]. Increased pain is a barrier to physical activity and exercise and may be related to the above-mentioned psychological factors [18]. A mental health treatment program tailored to these psychological risk factors, combined with an individually tailored education and exercise therapy program, could have the potential to improve outcome measures for patients with OA and patients undergoing TKA at increased risk of chronic pain and poor function following surgery. Hence, we designed an internet-delivered cognitive behavioral therapy (iCBT) program specially tailored to patients with OA and patients undergoing TKA [19] to be combined with exercise therapy. As advised by the UK Medical Research Council (MRC) we used their framework for developing and evaluating complex interventions [20]. The framework is particularly useful to ensure a systematic and thorough developing process before testing complex interventions in large resource-demanding randomized controlled trials (RCT), to avoid research waste. As recommended, we designed a feasibility study to identify uncertainties around recruitment and retention rate, as well as acceptability and expected adherence to the intervention itself.

The aim of this randomized feasibility trial was to investigate the feasibility of the intervention designed to improve outcomes for patients with knee OA and patients undergoing TKA at risk of poor outcomes after TKA and examine whether a three-armed RCT of such an intervention was feasible regarding (i) recruitment and retention rate, (ii) compliance to the intervention and follow-up, (iii) cross over and (iv) adverse events.

## Methods

### Study design

**Fig. 1:**
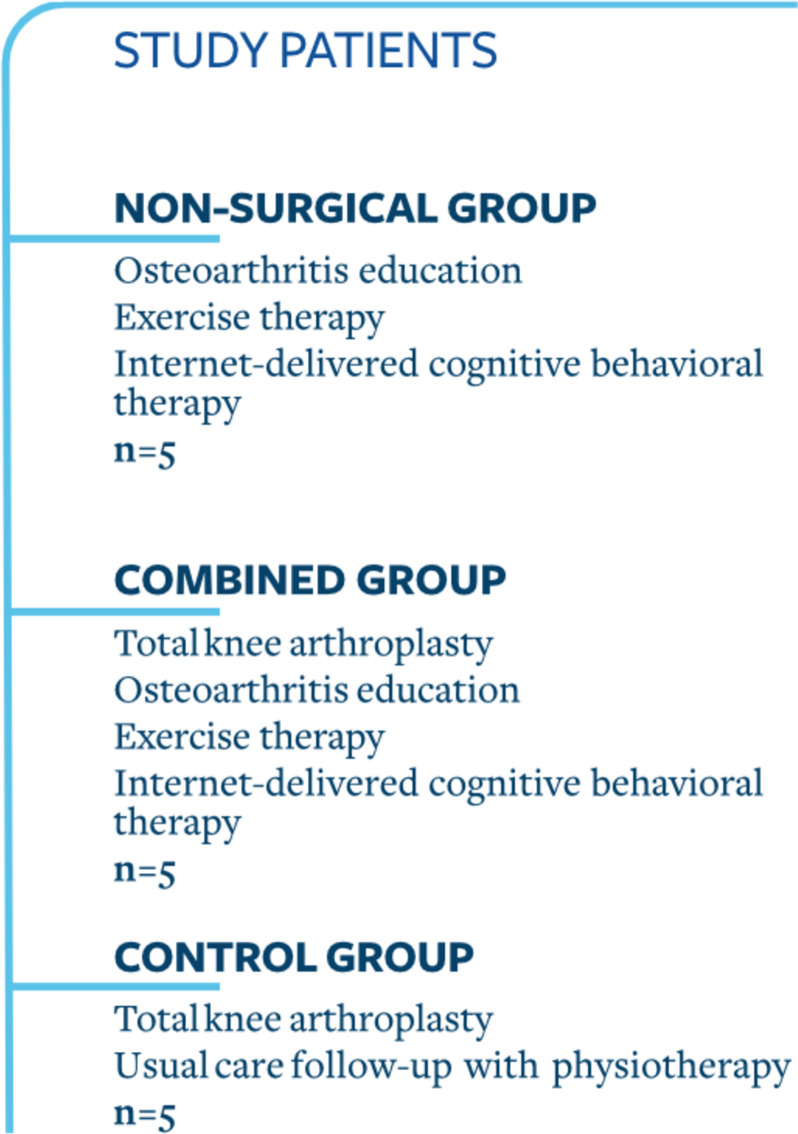
Groups in a randomized feasibility study for patients with knee osteoarthritis

We planned a three-armed multicenter RCT evaluating the effectiveness of a combined 12-week exercise therapy and education (ExE) program and a 10-module iCBT program delivered either alone (group A) or in combination with TKA (group B), compared to TKA with standard postoperative care (group C). This study is called The MultiKnee trial (Fig. 1). A randomized, three-armed, feasibility trial was conducted to assess the feasibility of such an RCT. The trial is reported according to the Consolidated Standards of Reporting Trials (CONSORT) statement extension to randomized pilot and feasibility trials [21].

### Participants

Patients with knee OA scheduled for primary TKA in two high-volume hospitals in the Western and Eastern parts of Norway between August 2019 and June 2020 were asked to participate. With this, we aimed for a sample consisting of patients from urban and rural areas of Norway thereby providing a geographically and socially diverse sample.

#### Eligibility criteria

Patients scheduled for primary TKA, aged ≥ 18, ≤ 80, with American Society of Anesthesiologists (ASA) grade 1–3, radiographic evidence of osteoarthritis (Kellgren-Lawrence grade 3 or 4), Body mass index (BMI) < 40 and were able to read and write in Norwegian, were to be recruited to the feasibility trial. Patients with previous unicompartmental or patellofemoral knee arthroplasty, large axis deviation or instability requiring the use of hinged prosthesis, diagnosis of dementia, or diagnosis of seropositive rheumatic disease, were excluded from the study.

#### Recruitment

Initially, we wanted to include only patients at risk of poor outcomes following TKA. All patients scheduled for TKA were screened for potential risk factors prior to eligibility assessment. The screening instrument used to identify patients at risk was based on prior studies on predictors of poor outcomes following TKA [22, 23] (Table 1). The evidence behind the instrument was weak. We therefore decided to include the screening questions in the baseline questionnaire, instead of including them in the eligibility criteria, so that the risk factors could be further assessed and evaluated.

**Table 1:** An overview of the screening instrument

	Instrument:	Cutoff^a^:
Age		< 55
Preoperative pain and function	The Knee Injury and OA Outcome Score (KOOS) pain and physical scale combined^b^	≤ 22
Widespread pain	Number of painful sites	≥ 2
Pain catastrophizing	Pain Catastrophizing Scale (PCS)^c^	≥ 30
Pain-related fear avoidance	Fear-Avoidance Belief Questionnaire (FABQ)^d^	> 14.9
Depression/anxiety	Hospital Anxiety and Depression Scale (HADS)^e^	> 11

^a^Scores below/above the cutoff point gave one point on each topic. Patients with two points or more were rated as at risk of poor outcomes and could be included in the study

^b^Higher score=more pain

^c^Higher score=more catastrophizing

^d^Higher score=more fear avoidance beliefs

^e^Higher score=more anxiety and depression

During a consultation at the outpatient clinic, the orthopedic surgeon assessed the patients for inclusion and exclusion criteria.

Eligible patients were thoroughly informed about the study, the randomization process, the interventions, and the possibility of withdrawing from the study. Patients who wanted to participate received an e-mail with a link to an electronic written consent form. The number of patients screened and reasons why potential participants were ineligible were recorded. Eligible patients who were approached but who declined to participate were anonymously recorded and the reason(s) for declining participation was recorded.

#### Randomization and blinding

Once the patients had signed the consent form, they were randomized by sealed opaque envelopes to one of three treatment groups in the ratio 1:1:1. The randomization scheme was computer-generated using permuted blocks of three or six, and the envelopes were prepared by an independent staff member and kept in a locked location. Patients in group A were referred to a physiotherapist for education and exercise therapy while patients in group B and C were scheduled for TKA.

Blinding of participants and physiotherapists who deliver the intervention was not possible due to the nature of the intervention.

### Intervention

**Fig. 2:**
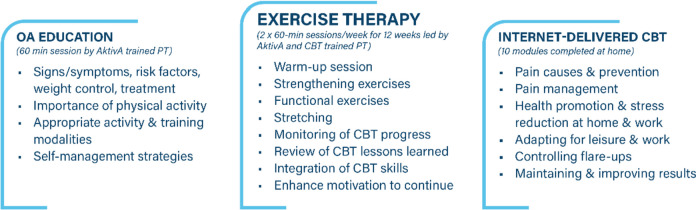
Overview of the MultiKnee program. Abbreviations: OA=osteoarthritis, CBT=Cognitive Behavioral Therapy, AktivA=active with osteoarthritis, PT=physiotherapist

The MultiKnee program is a combination of an individually tailored ExE program led by a physiotherapist, in addition to an iCBT program (Fig. 2). This complex intervention was to be tested in the MultiKnee trial. The ExE program is based on “AktivA”, which is an evidence-based and guideline-based implementation program to improve nonsurgical treatment for patients with knee OA in Norway [24]. Initially, we included only patients living near the hospitals to receive the exercise therapy at the hospitals. This largely limited the patient’s eligibility. We therefore extended the residential area nationwide, but the patients still had to be connected to the original two hospitals.

To standardize the intervention as much as possible, we preferred certified AktivA physiotherapists to deliver the ExE program. A study center AktivA physiotherapist delivered the education part of the program at the study center. However, a shortage of available AktivA-certified therapists resulted in the delay in the delivery of the 12-week exercise therapy program for several patients. We then also allowed physiotherapists without AktivA certification to deliver this part of the intervention. In such cases, the following was done to ensure standardization of the intervention: The AktivA certified study center physiotherapist contacted the non-certified physiotherapists and informed them thoroughly about the study and which principles to follow regarding pain management, dosage, and progression, and provided an informational leaflet including a selection of exercises with suggestions on individual tailoring and progression. Furthermore, the non-certified physiotherapists were contacted by the AktivA physiotherapist every second week to provide support and supervision through the intervention period. With these changes, we were able to recruit patients from across the country, ensuring that they received exercise therapy according to the AktivA principles.

#### Education

The education part of the intervention was based on the same as used in the AktivA program [24]. A study center AktivA certified physiotherapist led the patient education at one of the study centers, before the start of the exercise therapy program. A PowerPoint educational presentation was used by all physiotherapists to standardize the education sessions. The content of the educational part (the OA school) was developed based on previous published scientific papers, and clinical experience, and focused on updated knowledge about OA, risk factors, symptoms, managing life with OA, and possible treatment options. The beneficial effect of exercise on symptoms, physical function, and general health, and the effect of weight reduction and self-management strategies were highlighted. Patients were encouraged to engage and communicate, share experiences, identify possible obstacles, and discuss how to overcome them. The educational sessions lasted 60–90 min and were performed either in groups or individually depending on the number of participants in each clinic. Themes from the education session were discussed further by the physiotherapists during the exercise therapy and iCBT sessions.

#### Exercise therapy

The physiotherapy-guided AktivA program [24] was implemented individually or in group training sessions of 45–60 min × 2 per week for 12 weeks and individually adjusted with regard to dose and progression of exercises. The aim was to strengthen lower extremity muscles, increase range of motion (ROM), and improve balance and functional stability of the knee. Appropriate position of the joints, with hip, knee, and footwell aligned, was emphasized. The pain monitoring system described by Thomee in 1997 [25], was used. The pain was measured with a Visual Analog Scale (VAS) from 0 to 10 where zero is no pain and 10 is the worst possible pain. VAS 0-2 was considered safe and 2–5 was acceptable. If the patient experienced pain above five during or immediately after exercising, the exercises were adjusted. Pain should return to normal within 24 hours after exercise, if not, the dosage should be reduced.

#### Cognitive behavioral therapy

The iCBT program used in this trial is developed for, and targeted to improve pain and function for, patients with OA and patients undergoing TKA at risk of poor outcome [19] by targeting known psychological risk factors (i.e., anxiety, depression, and catastrophic thinking). The program was developed according to the first two steps in the Medical Research Council framework for complex interventions. The details of the program and its developmental process were previously published [19]. The program consists of 10 modules and a total of 86 tasks to be completed during the program. Each module follows a similar structure, with psychoeducational texts and videos presenting relevant topics, and tasks and exercises. A fictional character, receiving non-surgical or surgical treatment is presented and followed throughout the program. The theme and content for each module are presented in Table 2. In addition, the patients are mentored with telephone support sessions every second week, from the study center physiotherapists, trained by an experienced CBT psychologist. Furthermore, a manual was developed for the physiotherapists to ensure consistency. It contained the 10 modules from the iCBT program, and two extra learning modules developed specifically for the physiotherapists (modules 11 and 12). Module 11 introduced basic CBT and Motivational Interviewing (MI) principles, and module 12 provided guidance on how to handle patients’ resistance to the program and address potential challenges.

**Table 2:** The iCBT^a^ program, modules, themes, and content

Module	Theme	Content
1	Getting started	Introduction Port control theory Relaxation technique
2	Goals for the recovery	Five key elements important for coping with pain FAQ^b^ about exercise and activity Goals for recovery
3	Stress and pain	Change habits Stress and pain Locus of control
4	Lifestyle	How type of lifestyle can contribute to the symptoms Safety behaviour
5	Identifying automatic thoughts	Thinking errors Automatic thoughts The inner dialogue
6	Creating alternative thoughts	Twelve common thinking errors Generating alternative thoughts
7	Be more mindful	Default mode network and mental habits Focused attention
8	Selective attention	How to be more mindful Selective attention Unhelpful assumptions
9	Postponing worry and rumination	Worry and rumination How to make a postponement log
10	What’s next?	Summary What have you learned?
Learning modules for physiotherapists:		
11	Basic CBT^c^ for physiotherapists	Key elements for CBT
12	Talking to the patients	Motivational Interviewing techniques

^a^*iCBT*=internet-delivered cognitive behavioral therapy

^b^*iCBT*=internet-delivered cognitive behavioral therapy

^c^*CBT*=cognitive behavioral therapy

#### Standard postoperative care

Patients were mobilized to standing on the day of surgery whenever possible, and full weight bearing on the operated knee was permitted. Standardized physiotherapy, including both active and passive flexion and extension exercises, was initiated on the day after surgery. Patients used crutches for mobilization and were typically discharged on the second-day post-surgery. Within 2 weeks after discharge, patients in group B started the MultiKnee program. Patients in group C received standard care physiotherapy in the municipalities, typically involving exercise therapy with varying levels of supervision, aimed at improving range of motion, strength, balance, and gait.

### Outcomes and statistics

Three main changes were performed in the recruitment process: (1) screening all patients for risk factors from the middle of August 2019 to the beginning of December 2019. (2) Screening only candidates for TKA for risk factors from the beginning of December 2019 to the middle of March 2020. (3) No screening for risk factors from April 2020 to July 2020. Numbers and percentages describe the recruitment rate.

Compliance with the intervention was reported as the number of compilers for each of the treatment options. Treatment compliance was defined as acceptable when patients had attended at least 75% of the exercise therapy sessions and had completed at least 75% of the iCBT tasks.

Outcome measures included both Norwegian versions of Patient Reported Outcome Measures (PROM) [26] and physical-performance tests (Table 3) and are described as numbers of patients who completed the PROMs and physical-performance tests at baseline and at 3-, 6- and 12 months after the start of the intervention. Crossovers are reported as numbers of patients who crossed over from one group to another within the first year.

**Table 3:** Patient-reported outcome measures and clinical assessments

	Baseline	3 months	6 months	12 months
Patient-reported outcome measures (PROM)				
1. Socio-demographics	x			
2. Self-reported comorbidity	x			
3. Health-related Quality of life (EQ-5D-5L)	x	x	x	x
4. Brief Pain Inventory (BPI)	x	x	x	x
5. Knee Injury and Osteoarthritis Outcome Score (KOOS)	x	x	x	x
6. Forgotten Joint Score (FJS-12)	x	x	x	x
7. Fear-Avoidance Belief Questionnaire (FABQ)	x	x	x	x
8. Pain Catastrophizing Scale (PCS)	x	x	x	x
9. Patient-acceptable symptom state (PASS)		x	x	x
10. Treatment failure		x	x	x
11. Global Perceived Effect (GPE)		x	x	x
12. Locus of Control Scale	x	x	x	x
13. Pittsburgh Sleep Quality Index	x	x	x	x
14. Physical activity (SoC^a^, HUNT^b^ 2)	x	x	x	x
15. Hospital Anxiety and Depression Scale (HADS)	x	x	x	x
Clinical assessments				
16. ActiGraph GT3X-BT Activity monitor	x		x	x
17. The 40-meter Fast-paced Walk Test	x	x	x	x
18. The Stair Climb Test	x	x	x	x
19. 30-second sit-to-stand test	x	x	x	x
20. Range of Motion (ROM)	x	x	x	x
21. Body Mass Index (BMI)	x	x	x	x
22. X-rays	x			x

^a^*SoC* State of change physical activity

^b^*HUNT* Nord-Trøndelag health study

Adverse events and serious adverse events were registered in three steps: screening of the medical records at the hospitals, reports by the physiotherapists, and self-reported by the patients, using questionnaires. Medical records were screened at 12 months for all adverse events from inclusion until the 12-month follow-up. An adverse event was defined as any undesirable experience during follow-up that led to contact with the health care system. A serious adverse event was defined as any event that led to hospitalization, prolonged in-hospital care or additional surgery, was life-threatening or resulted in permanent disability or damage, or death [27]. Numbers and types of adverse events were described.

Demographic characteristics are reported in mean and standard deviation (SD). The analysis of clinical outcome measures was descriptive and reported as median and interquartile ranges (IQR).

### Sample size

The sample size for this feasibility study was based on practical considerations, budgetary constraints, and the number of participants needed to reasonably evaluate the feasibility goals, as recommended by the National Center for Complementary and Integrative Health (NCCIH) [28]. In its nature, this work is qualitative and descriptive, and we did not aim to evaluate group differences or effect sizes. Thus, a sample of less than 30 may be adequate [29]. For this complex trial, we considered that 5 participants per group would be sufficiently large to inform our research questions, and realistic given our timeline.

The sample size for the full-scale trial was revised as a result of the feasibility trial. Before the feasibility trial, the sample size was estimated to be 62 patients per group, allowing for a 20% dropout we would need 223 patients. The revised sample size was based on an estimated minimal clinical perceptible improvement of 10 points in the primary outcome KOOS. Based on a previous study, we set the standard deviation of change to 16 [30]. This revised calculation revealed that we would need 78 patients in each treatment group. To allow for a 20% dropout, 282 patients will be recruited in the full-scale trial. The details of sample size estimation are described elsewhere [26].

## Results

**Fig. 3:**
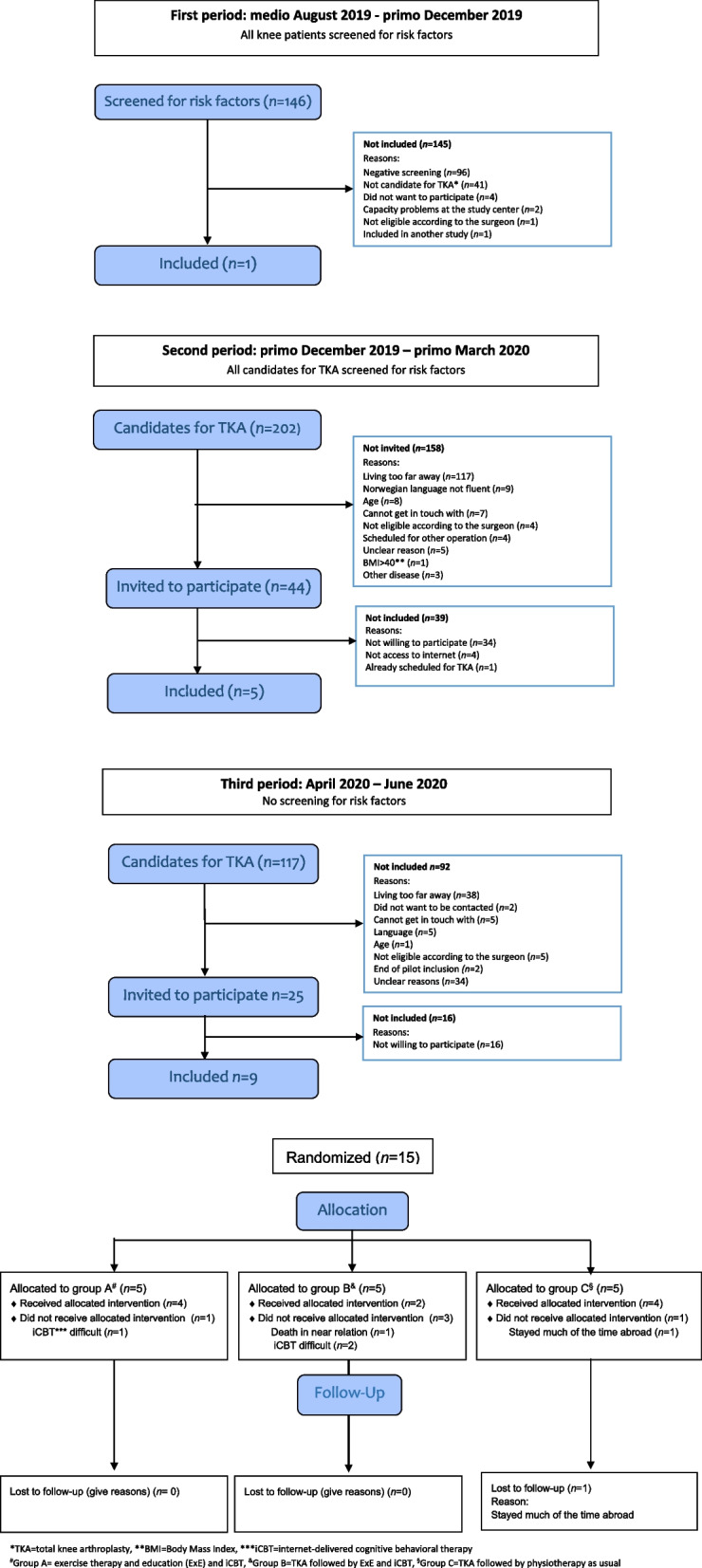
Flow diagram for a randomized feasibility study for patients with knee osteoarthritis

Between August 2019 and June 2020, 350 patients were assessed for eligibility. Fifteen patients were included in the feasibility study and randomized into three groups. The inclusion of patients and attrition at follow-up is shown as a flow diagram in Fig. 3. Demographic characteristics of the patients included are shown in Table 4.

**Table 4:** Demographics in a randomized feasibility study for patients with knee osteoarthritis^a^

	Group A^b^ (*n* = 5)	Group B^c^ (*n* = 5)	Group C^d^ (*n* = 5)	Total (*n* = 15)
Female sex—*n*	3	3	2	8
Age—years	61.6 ± 6.19	63.8 ± 7.19	66.0 ± 9.08	63.8 ± 7.26
Weight—kg	81.4 ± 7.60	86.3 ± 8.08^e^	97.5 ± 12.24^f^	88.5 ± 11.99^g^
Body mass index—kg/m^2^	28 ± 2.64	29 ± 2.58 ^e^	32 ± 4.4^f^	30 ± 3.6^g^

^a^Plus-minus values are means±SD

^b^Group A=exercise therapy and education(ExE)+internet-delivered cognitive behavioral therapy (iCBT)

^c^Group B=total knee arthroplasty (TKA) followed by ExE+iCBT

^d^Group C=TKA followed by physiotherapy as usual

^e^*n=3*

^f^*n=4*

^g^*n=9*

### Recruitment of participants

The recruitment process is thoroughly described in three time periods based on the changes we made (Fig. 3).

- 1) Screening all patients for risk factors from the middle of August 2019 to the beginning of December 2019.

This screening procedure was found too demanding for both the study staff and the patients. Many patients who did not fulfill the other inclusion criteria, such as indication for surgery, were screened for risk factors to no avail.

- 2) Screening only candidates for TKA for risk factors from the beginning of December 2019 to the middle of March 2020.

The recruitment rate remained too low to justify initiating a large RCT, indicating that the screening algorithm might be too strict and reduced the number of potential candidates significantly. However, a less strict screening algorithm may lead to higher imprecision. Thus, the research team decided to include all patients who were candidates for TKA and instead incorporate the screening questionnaire into the baseline questionnaire.

At this time, the country was locked down due to the COVID-19 pandemic, and recruitment to the study was paused for 2 months.

- 3) No screening for risk factors from May 2020 to July 2020*.*

Since the major reason for not being included was living too far away, we changed this criterion at the end of May 2020. Recruitment for the pilot study was completed on June 10, 2020.

### Compliance with the intervention and follow-up

In total, nine out of 10 completed at least 75% of the ExE sessions, six out of 10 patients completed at least 75% of the iCBT program, and nine out of 10 underwent TKA surgery.

#### Education

All patients in groups A and B attended the education session at one of the study hospitals.

#### ExE

All five patients in the non-surgical group A attended all ExE sessions. In surgical group B, four patients attended all ExE sessions; one patient attended 12 sessions (50%) due to bereavement. In total, 9 out of the 10 participants completed at least 75% of the ExE program and were defined as compliers.

#### iCBT

One patient, included early in the study period, received the prototype version of the program with 12 modules and 113 tasks. This patient completed 26 of 113 tasks (32%). The other nine patients received the second version of the program with 10 modules and 86 tasks. They completed a mean of 68 out of 86 tasks (79%). Six out of 10 patients completed more than 75% of the tasks.

In total, patients in group A completed 83% of the tasks, four out of five patients completed more than 75% of the tasks, one patient thought the program was too demanding and completed 60% of the tasks. In total, all patients in group B completed 57% of the tasks, one patient got the prototype version of the program with more tasks. One had back problems and was unable to sit by the computer, and one experienced death in near relation. Two out of five patients in group B completed more than 75% of the iCBT program.

#### Follow-up

Fourteen out of 15 patients answered the baseline and 3-month questionnaire, 13 answered at 6 months and 12 months. Median and IQR for the key outcomes are presented in Table 5.

**Table 5:** Outcome in PROMS data and clinical examination

			Baseline			3 months			6 months			12 months	
	Group^a^	*N*	Median	IQR^b^	*N*	Median	IQR	*N*	Median	IQR	*N*	Median	IQR^a^
KOOS^c^ pain	A	5	41.67	31.94	5	62.89	26.39	5	66.67	23.61	4	62.50	17.36
	B	5	47.22	25.00	5	75.00	30.56	5	88.56	26.39	5	88.89	23.61
	C	4	44.44	54.86	4	62.50	34.03	3	66.67		4	80.56	49.31
KOOS symptoms	A	5	50.00	26.79	5	71.43	26.79	5	67.86	30.36	4	69.64	32.14
	B	5	60.71	12.50	5	60.71	28.57	5	78.57	30.36	5	85.71	12.50
	C	4	50.00	55.36	4	66.07	35.71	3	53.57		4	73.21	38.39
KOOS adl^d^	A	5	54.41	33.82	5	76.47	20.59	5	75.00	33.09	4	69.12	28.68
	B	5	57.35	38.24	5	83.82	21.32	5	91.18	22.06	5	94.12	12.50
	C	4	52.94	46.69	4	70.59	33.82	3	64.71		4	83.82	54.78
KOOS sport/recreation	A	5	15.00	27.50	5	35.00	20.00	5	20.00	32.50	4	35.00	17.50
	B	5	25.00	32.50	5	35.00	20.00	5	35.00	17.50	5	50.00	22.50
	C	4	20.00	25.00	4	37.50	22.50	3	30.00		4	35.00	40.00
KOOS qol^e^	A	5	37.50	40.63	5	50.00	37.50	5	50.00	28.13	4	50.00	40.63
	B	5	37.50	15.63	5	68.75	21.88	5	62.50	25.00	5	87.50	18.75
	C	4	34.38	39.06	4	56.25	31.25	3	50.00		4	59.38	48.44
PCS^f^ total	A	5	10.00	3.50	5	18.00	18.00	5	5.00	5.00	4	5.50	7.25
	B	4	10.50	14.50	5	18.00	3.00	5	3.00	9.00	5	2.00	1.50
	C	4	18.00	29.25	4	19.50	13.25	3	8.00		4	8.50	16.25
HADS^g^ anxiety	A	5	5.00	2.00	5	2.00	5.00	5	3.00	3.50	4	2.00	2.00
	B	3	7.00		5	3.00	3.50	5	3.00	3.00	5	3.00	3.50
	C	4	4.00	8.00	4	2.50	4.50	3	2.00		4	0.50	7.00
HADS depression	A	5	3.00	3.00	5	2.00	3.00	5	1.00	1.50	4	1.50	4.00
	B	3	3.00		5	2.00	2.50	5	1.00	1.00	5	2.00	1.50
	C	4	4.00	7.25	4	3.50	7.50	3	3.00		4	3.00	8.50
HADS sum	A	5	8.00	2.00	5	6.00	6.00	5	4.00	5.00	4	3.50	5.50
	B	3	10.00		5	5.00	4.00	5	4.00	2.00	5	3.00	3.00
	C	4	8.00	15.25	4	6.00	12.00	3	4.00		4	3.50	15.00
FJS^h^	A	5	12.50	35.42	5	22.91	23.96	5	22.91	25.00	4	33.33	16.15
	B	5	12.50	33.33	5	39.58	65.63	5	64.58	40.62	5	64.58	35.42
	C	4	19.79	17.71	4	22.91	38.54	3	27.08		4	27.08	72.92
FABQ^i^	A	5	6.00	7.00	5	5.00	6.00	5	4.00	6.00	4	6.00	5.25
	B	5	14.00	15.50	5	3.00	6.50	5	0.00	1.50	5	2.00	6.00
	C	4	18.00	10.75	4	9.50	10.00	3	10.00		4	10.00	18.00
Stair test (s)	A	5	9.94	3.85	5	8.32	2.27	5	9.16	2.67	5	8.53	1.43
	B	5	10.97	8.82	5	10.22	5.55	4	9.19	3.20	5	8.22	5.02
	C	4	12.89	31.13	4	18.10	9.27	4	15.58	12.59	4	14.88	16.89
Sit to stand	A	5	20.00	6.50	5	24.00	6.50	5	22.00	12.50	5	24.00	9.50
	B	5	13.00	5.00	5	17.00	6.00	4	16.50	4.75	5	21.00	11.00
	C	4	12.50	10.50	4	11.50	6.25	4	11.50	10.25	4	14.50	10.50
40-m walk test (s)	A	5	19.24	7.35	5	19.37	6.84	5	18.75	3.93	5	18.33	5.47
	B	5	27.73	5.63	5	23.37	6.69	4	21.53	7.61	5	18.21	10.13
	C	4	25.93	44.20	4	27.03	7.69	4	28.09	13.05	4	27.58	26.00
Active flexion	A	5	130.00	10.00	5	125.00	12.50	5	125.00	15.00	5	120.00	7.50
	B	5	120.00	10.00	5	110.00	2.50	4	112.50	12.50	5	115.00	22.50
	C	4	122.50	23.75	4	117.50	12.50	4	110.00	17.50	4	115.00	10.00
Active extension	A	5	− 5.00	10.00	5	0.00	15.00	5	− 5.00	7.50	5	− 5.00	5.00
	B	5	− 10.00	10.00	5	− 10.00	10.00	4	− 5.00	7.50	5	0.00	7.50
	C	4	− 17.50	12.50	4	− 7.50	8.75	4	− 6.00	8.00	4	− 7.50	8.75

^a^*Group A* MultiKnee program, *Group B* total knee arthroplasty followed by the MultiKnee program, *Group C* total knee arthroplasty followed by physiotherapy as usual

^b^*IQR* interquartile range

^c^*KOOS* Knee Injury and Osteoarthritis Outcome Score 0–100-higher score=less problems

^d^*adl* activity of daily living

^e^*qol* quality of life

^f^*PCS* Pain Catastrophizing Scale—higher score=more catastrophizing

^g^*HADS* Hospital Anxiety and Depression Scale—higher score=more anxiety and depression

^h^*HADS* Hospital Anxiety and Depression Scale—higher score=more anxiety and depression

^i^*FABQ* Fear-Avoidance Belief Questionnaire—0–24-higher score=more fear avoidance beliefs

Fourteen patients completed the physical performance tests at baseline and at 3-, 6-, and 12 months (Table 6).

**Table 6:** Data completeness for physical performance tests from baseline to 1 year, *n* (%)

	Baseline *n* (%)	3 months *n* (%)	6 months *n* (%)	12 months *n* (%)
Group A^*^ *n* = 5	5 (100)	5 (100)	5 (100)	4 (80)
Group B^a^ *n* = 5	5 (100)	5 (100)	4 (80)	5 (100)
Group C^b^ *n* = 5	4 (80)	4 (80)	4 (80)	4 (80)
Total *n* = 15	14 (93)	14 (93)	13 (87)	13 (87)

*Group A=exercise therapy and education(ExE)+internet-delivered cognitive behavioral therapy (iCBT)

^a^Group B=exercise therapy and education(ExE)+internet-delivered cognitive behavioral therapy (iCBT)

^b^Group C=TKA followed by physiotherapy as usual

### Cross over

No participants crossed to surgery within the first year.

### Adverse events

One participant in group B experienced hyperesthesia in part of the scar, treated by the surgeon with local anesthesia and cortisone. No other adverse events were registered.

## Discussion

The lessons learned in this feasibility study were crucial to refine the procedures, as well as the acceptability of the complex intervention itself, prior to testing and evaluating the intervention in a future RCT. In particular, the feasibility study provided critical insights into serious threats to the recruitment rate.

These insights resulted in important changes for the improvement of the recruitment strategy for the ongoing MultiKnee RCT [26].

### Recruitment and retention rate

Challenges regarding recruitment to surgical trials are common and have been described in other studies [31, 32]. Initially, we attempted to recruit patients at risk for a poor outcome based on screening using the validated appropriateness classification system developed by Escobar et al. [22] and studies on risk factors. However, our screening tools’ accuracy in identifying patients at higher risk for a poor outcome had not been evaluated, and the low recruitment rate indicated that it was not reliable enough to identify patients relevant to the study. We therefore decided to integrate the risk factors into the baseline questionnaire and stop recruiting based on the risk factors. To ensure sufficient statistical power to identify significant differences between groups in the full-scale trial, a new sample size estimation was performed to account for a revised sample, including patients with and without a higher risk for a poor outcome. This change in the inclusion criteria increased the recruitment rate significantly.

Preference for either surgical or non-surgical treatment was considered a potential threat to inclusion in our study that we wanted to test in the feasibility trial. In a qualitative synthesis study, Davies and colleagues [33] found that many patients and healthcare professionals had a strong preference for either surgery or non-surgical treatment. Preoperative decision-making is a complex process for both clinician and patient. Despite the large number of knee replacements undertaken, no clear consensus exists within the surgical community about exact indications, particularly in terms of severity of preoperative symptoms, obesity, and age [34]. Based on our experiences in the recruitment process, it is essential to inform patients in a way that they understand the pros and cons of each treatment to be able to make a well-informed choice regarding trial participation. Recruitment of patients to the study depends on the surgeons’ participation. Training and support can make them more comfortable in the recruitment process [35].

As the guidelines state that exercise therapy is the first-line treatment for patients with OA, many patients may have tried this before they were referred to the orthopedic surgeon. However, Bruhn et al [36] found in their study that only 41% of patients had received supervised land-based exercise, and 23% of patients had participated in patient education prior to consultation with the orthopedic surgeon. Some of the patients in the current study may have declined to participate because they had already attended exercise therapy programs similar to the exercise program in this study.

In the recruitment process, when informing patients about the study, some patients decided to decline randomization which could lead to being randomized to surgery, as they had not tried the supervised exercise of sufficient dose and length first.

### Compliance with the intervention and follow-up

We found that compliance with the ExE program was higher than compliance with the iCBT program. Some patients found it hard to understand how a psychological intervention could help their knee problems whereas the rationale behind exercise therapy seemed easier to understand.

The iCBT program was therefore revised and shortened to be more accessible, relevant, and understandable during the feasibility study [19]. This cyclic process of refinement included tailoring the intervention even more to patients with OA and patients undergoing TKA, simplifying the language, and making navigation in the program easier, in line with the MRC framework [20] before implementing it in the definite RCT.

### Crossover

Patients in non-surgical group A were asked to delay the operation for at least 1 year. Although we had anticipated a potential risk that some patients would decide to undergo TKA surgery before a year had passed, no one crossed over during the first year. This may be due to the small sample size in this study. Skou and colleagues [4] reported that 26% crossed over from non-surgical to surgical group within the first year. Some precautions can be made to reduce crossover or discontinuation. In-depth information about the study and its implications for the participants is crucial. Informational videos can be a valuable supplement to oral and written information [37].

### Adverse events

All surgical procedures involve a potential risk of serious adverse events [38]. No serious events were registered in this study. This is most likely due to the small sample size. Skou and colleagues found that the incidence of adverse events was higher in the surgical group than in the non-surgical group [4].

### Strengths and limitations

Randomized controlled trials (RCT) are expensive and time-consuming endeavors. To avoid waste in research, developing studies with high methodological quality has been highlighted [39], which was especially relevant in the process of developing this complex intervention trial. Our study illustrates the importance of following a systematic process including feasibility testing, as recommended in the MRC framework [20]. The complex intervention in this trial is a strategic selection of treatment modalities. The ExE program is based on AktivA, a well-documented program based on international guidelines for the nonsurgical treatment of patients with OA in Norway [24]. Similar models have been in use in Sweden (BOA) [40] since 2008 and in Denmark (GLA:D) [41] since 2013, and these programs have shown to be well suited for clinical practice and results show significant improvements concerning pain, physical function, and health-related quality of life in patients with hip and/or knee osteoarthritis [41, 42]. In addition, the neuromuscular exercise program used in GLA:D has previously been shown to be effective for patients with moderate to severe osteoarthritis eligible for TKA and after undergoing TKA [15].

The iCBT program has been through a thorough development process, following the UK Medical Research Council framework for developing complex interventions [20]. By conducting a feasibility trial, we ensure the feasibility of a future RCT, and that the intervention is relevant and acceptable for its target group. The refining of the iCBT program will probably increase compliance with the intervention. The feasibility trial was not powered to investigate the effect of the intervention. The ongoing RCT will provide valuable information on the potential this treatment has to improve outcomes in knee OA and TKA patients [26].

The sample size in this study was small, which is a limitation. However, the feasibility trial was not intended to have the power to investigate the effect of the intervention. Therefore, we believe that a sample of 15 patients was sufficient to address our research questions.

The inclusion criteria in this study can have resulted in reduced generalizability. Recruitment of patients was conducted at two hospitals in different parts of Norway. This ensured participants both from urban and rural areas in Norway. The criteria for Norwegian writing and reading competence can have excluded a portion of the OA and TKA patients with other native languages.

We limited our pool by including only patients with a combination of radiographic and clinical manifestations of OA. Because 2 of 3 participants were randomized to surgery, we needed to be sure that all patients had radiographic changes compatible with OA. Without such changes, there would not be an indication for surgery, and it would be unethical to allow them to undergo surgery. Because of this, our findings may not be generalizable to patients with low-grade radiographical OA.

A feasibility study has an important role in designing an RCT [43]. Our study revealed weaknesses in the recruitment process and possible threats to patients’ compliance with the intervention. The adjustments made on the inclusion and exclusion criteria were crucial to ensure an appropriate recruitment rate and will strengthen the planned RCT. This study illustrates the importance of evaluating the feasibility of complex interventions in terms of recruitment procedures, retention rate, and acceptability of the intervention, as suggested by the MRC framework for complex interventions [20]. Findings from this study resulted in further development and improved feasibility of our protocol, thus leading to a feasible and well-managed full-scale RCT [26].

## Conclusions

The findings from this study suggested that it was feasible to conduct a definite and methodologically robust RCT evaluating the effectiveness of a combined education, exercise therapy, and cognitive behavioral therapy program in patients with osteoarthritis eligible for TKA, either instead of or in addition to TKA. The recruitment process was challenging initially and several changes during the study were necessary to increase recruitment. While compliance with the education, exercise therapy, and follow-up was high, revision of the developed iCBT program was necessary to increase compliance.

## Data Availability

Due to Norwegian ethical and legal restrictions, the dataset generated and analyzed during the current study is stored at the Service for Sensitive Data (TSD) at the University of Oslo. Requests for access to an anonymized data set can be sent to Anners Lerdal (anle@lds.no). Requests must specify what the data will be used for, who will be responsible for storage, and how it will be stored. Final approval from the Data Protection Officer and the Regional Committees for Medical and Health Research Ethics will be required prior to the release of the anonymized minimal dataset.

## Abbreviations

CONSORT: The Consolidated Standards of Reporting Trials
ExE: Exercise therapy and education
iCBT: Internet-delivered cognitive behavioral therapy
IQR: Inter-quartile range
MI: Motivational interviewing
OA: Osteoarthritis
OARSI: The Osteoarthritis Research Society International
PROM: Patient reported outcome measures
RCT: Randomized controlled trial
ROM: Range of motion
TKA: Total knee arthroplasty
VAS: Visual analog scale
